# ZFP36L1 promotes monocyte/macrophage differentiation by repressing CDK6

**DOI:** 10.1038/srep16229

**Published:** 2015-11-06

**Authors:** Ming-Tai Chen, Lei Dong, Xin-Hua Zhang, Xiao-Lin Yin, Hong-Mei Ning, Chao Shen, Rui Su, Feng Li, Li Song, Yan-Ni Ma, Fang Wang, Hua-Lu Zhao, Jia Yu, Jun-Wu Zhang

**Affiliations:** 1 The State Key Laboratory of Medical Molecular Biology and the Department of Biochemistry and Molecular Biology, Institute of Basic Medical Sciences, Chinese Academy of Medical Sciences and Peking Union Medical College, Beijing 100005, China; 2 Haematology Department, the 303 Hospital, Nanning, China; 3 Department of Hematopoietic Stem Cell Transplantation, Affiliated Hospital to Academy of Military Medical Science, Beijing, China

## Abstract

RNA binding proteins (RBPs)-mediated post-transcriptional control has been implicated in influencing various aspects of RNA metabolism and playing important roles in mammalian development and pathological diseases. However, the functions of specific RBPs and the molecular mechanisms through which they act in monocyte/macrophage differentiation remain to be determined. In this study, through bioinformatics analysis and experimental validation, we identify that ZFP36L1, a member of ZFP36 zinc finger protein family, exhibits significant decrease in acute myeloid leukemia (AML) patients compared with normal controls and remarkable time-course increase during monocyte/macrophage differentiation of PMA-induced THP-1 and HL-60 cells as well as induction culture of CD34^+^ hematopoietic stem/progenitor cells (HSPCs). Lentivirus-mediated gain and loss of function assays demonstrate that ZFP36L1 acts as a positive regulator to participate in monocyte/macrophage differentiation. Mechanistic investigation further reveals that ZFP36L1 binds to the CDK6 mRNA 3′untranslated region bearing adenine-uridine rich elements and negatively regulates the expression of CDK6 which is subsequently demonstrated to impede the *in vitro* monocyte/macrophage differentiation of CD34^+^ HSPCs. Collectively, our work unravels a ZFP36L1-mediated regulatory circuit through repressing CDK6 expression during monocyte/macrophage differentiation, which may also provide a therapeutic target for AML therapy.

Approximately two-thirds of protein abundance variation of mammalian cells can be accounted for by post-transcriptional mechanisms^1^. Accumulating evidence suggests that post-transcriptional regulation plays a critical role in many biological processes and a wide spectrum of pathologies^2^,^3^,^4^. MicroRNAs (miRNAs) and RNA binding proteins (RBPs) are emerging as two key determinants of post-transcriptional control^5^. MiRNAs are typically small (~22nt), non-protein-coding RNAs that negatively regulate genes expression at the post-transcriptional level. They mainly bind to the 3′untranslated regions (UTRs) of targets and specifically cleavage the target mRNAs while it is perfectly complementary to the mRNAs or repress the translation if no sufficient complementary sites exist^6^. RBPs are proteins that bind to the double or single-stranded RNA with their RNA binding domains and participate in forming ribonucleoprotein (RNP) complexes to influence RNA metabolism^7^. Eukaryotic cells encode diverse RBPs. Approximately 1000 genes have been annotated in RBP DataBase (RBPDB), with unique RNA-binding activity and protein-protein interaction^8^. RBPs nearly control every fate of the transcripts after transcription, such as alternative splicing, modification, export, localization, stability and translation^9^. With the development and wide-spread application of transcriptome sequencing, RBPs and ncRNAs mediated regulatory network has been intensively studied and relevant databases have been developed to annotate the interactions between RBPs and RNAs^10^,^11^,^12^.

RNA binding protein ZFP36L1, also known as TIS11B, is a member of ZFP36 zinc finger protein family of the early response gene induced by the phorbol ester 12-O-tetradecanoylphorbol-13-acetate (TPA) in murine fibroblasts. In mammalians, the family contains two other members called ZFP36/TIS11 and ZFP36L2/TIS11D. In addition to these well-described members, rodents possess an additional member officially named as ZFP36L3 specifically expressed in the placenta^13^,^14^. All members of the family are characterized by the presence in their coding sequence of a very particular tandem zinc-finger domain (TZF) which is composed of a double zinc-finger motif of the Cys-Cys-Cys-His (CCCH) type and each of the motifs is preceded by a leader sequence and bears RNA binding activity^15^. In the past few decades, all the three members have been extensively investigated in cell models and primary cells, which implicated their pro-apoptotic function and involvement in cell differentiation through a variety of mechanisms^16^. However, the three members mainly bind to the adesine-uridine (AU) rich elements (AREs) in the 3′UTRs of mRNAs leading to the target RNA destabilization. ZFP36L1 negatively regulates VEGF and BCL2 mRNA expression by binding to the AREs in their 3′UTRs^17^,^18^. ZFP36 inhibited TNF-alpha production from macrophages by destabilizing its messenger RNA, which also resulted from direct ZFP36 binding to the AREs of the TNF-alpha mRNA^19^. ZFP36L1 and ZFP36L2 specifically bind to the 3′UTR of LDLR mRNA and recruit the CCR4-NOT-deadenylase complex, resulting in mRNA degradation^20^. Definitive insight into the distinct role of ZFP36 family members has been provided by gene targeting studies in mice. Deletion of *ZFP36* leads to a spontaneous, non–cell-autonomous granulocyte hyperplasia, which strongly resembles reactive granulopoiesis with the elevated levels of inflammatory cytokines^21^. ZFP36L2 knockout (KO) mice exhibit defective hematopoiesis. Homozygous *ZFP36L2* KO mice died within approximately 2 weeks of birth, apparently from intestinal or other hemorrhage. Analysis of peripheral blood from ZFP36L2 KO mice showed a decrease in red and white blood cells, hemoglobin, hematocrit and platelets, which implies ZFP36L2 as a critical regulator of hematopoiesis^22^. Double KO of *ZFP36L1/L2* impairs mouse thymic development and leads to T lymphoblastic leukemia with deregulated notch pathway^23^.

Hematopoiesis is a highly orchestrated process which involves the expansion and differentiation of the limited number of hematopoietic stem cells (HSCs) into multipotential and lineage-committed progenitors, leading to the production of mature and functional blood cells^24^. The process is elaborately controlled by a complex regulatory network including RBPs. Over-expression of ZFP36L1 and ZFP36 in CD34^+^ hematopoietic progenitors impairs erythroid differentiation by mediating stat5b mRNA degradation through binding to its 3′UTR^25^. Hou, V.C. *et al.* reported that hnRNPA1-mediated protein 4.1R exon 16 (E16) splicing is required for the establishment of proper mechanical integrity of the erythrocyte membrane during erythropoiesis^26^. Hu *et al.* characterized a Cpeb4-mediated negative translational regulatory circuit that is required for terminal erythropoiesis^27^. However, RBPs-mediated post-transcriptional regulation of granulocytic differentiation and monocyte/macrophage differentiation is less known. Here in this study, through bioinformatics analysis and subsequent experimental validation, we found that ZFP36L1 expression was aberrantly decreased in acute myeloid leukemia (AML) patients compared with normal controls and selectively up-regulated during monocyte/macrophage differentiation and facilitated the process by directly binding to AREs in the 3′UTR of CDK6 mRNA, leading to decreased expression of CDK6, which unravels a RBP-mediated regulatory circuit composed of ZFP36L1 and CDK6 and provides a potential target for AML therapy.

## Results

### Bioinformatics analysis and experimental validation to screen potential RBPs involved in myeloid differentiation

**Figure 1:**
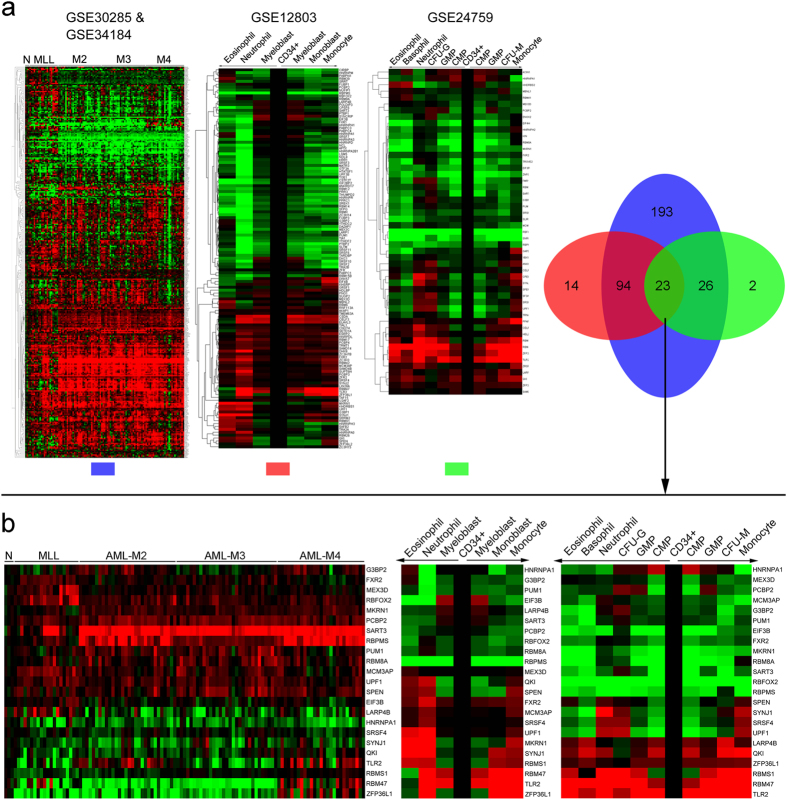
Bioinformatics analysis to screen potential RBPs involved in myeloid differentiation. (**a**) RBPs with differential expression in AML patients and myeloid differentiation were analyzed using array data (GSE30285, GSE34184, GSE12803 and GSE24759) annotated in GEO DataSets. (**b**) Twenty-three RBPs showed differential expression in both AML patients compared with normal controls and during the *in vitro* myeloid differentiation of CD34^+^ HSPCs.

**Figure 2:**
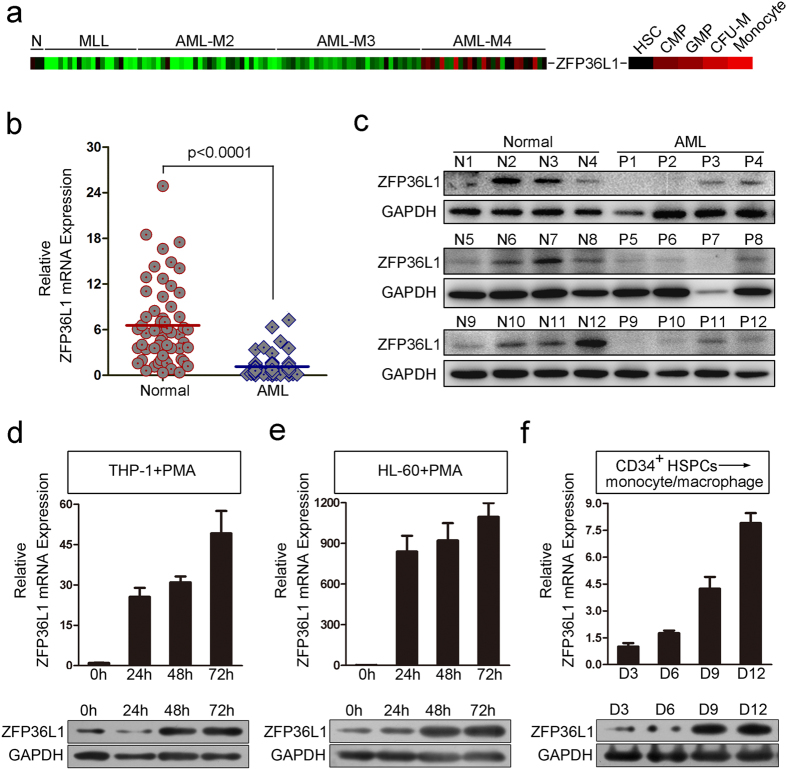
ZFP36L1 is validated as a potential regulator in monocytic differentiation. (**a**) Expression profile of ZFP36L1 in AML patients and monocytic differentiation derived from the array data. (**b**) Real-time PCR analysis of ZFP36L1 mRNA expression in peripheral blood MNCs derived from 56 AML patients (FAB M1 to M5 subtypes) and 57 normal controls. (**c**) Western blot analysis of ZFP36L1 protein level in 12 AML patients and 12 normal controls. (**d**) Real-time PCR and western blot analyses of ZFP36L1 expression during PMA-induced monocyte/macrophage differentiation of THP-1 cells. (**e**) Real-time PCR and western blot analyses of ZFP36L1 expression during PMA-induced monocyte/macrophage differentiation of HL-60 cells. (**f**) Real-time PCR and western blot analyses of ZFP36L1 expression during the *in vitro* monocytic induction culture of CD34^+^ HSPCs.

To systematically screen the RBPs involved in myeloid differentiation, we performed bioinformatics analysis using the expression profiling data of AML patients (GSE30285 and GSE34184) and *in vitro* myeloid differentiation from hematopoietic stem/progenitor cells (HSPCs) (GSE12803 and GSE24759) annotated in the GEO DataSets. We first analyzed the differentially expressed genes in AML patients compared with the normal controls and during the *in vitro* myeloid differentiation of CD34^+^ HSPCs, and then mapped the genes to the RBP DataBase, and finally got the differentially expressed RBPs (Fig. 1a, left). Twenty-three RBPs with differential expression in both AML patients and myeloid differentiation were screened (Fig. 1a, right) and their expression spectrum were presented separately (Fig. 1b). Next, we chose some of the 23 RBPs for experimental validation in AML patients with health donors used as normal controls and phorbol myristate acetate (PMA)-induced monocytic differentiation of THP-1 and HL-60 and all transretinoic acid (ATRA)-induced granulocytic differentiation of HL-60 and NB-4 as well as *in vitro* induction culture of CD34^+^ HSPCs derived from human umbilical cord blood (UCB). Finally we focused on ZFP36L1 whose expression profile (derived from the array data) in AML patients and monocytic differentiation was presented as (Fig. 2a). Besides, the experimental validation results also unraveled that ZFP36L1 expression exhibited significant down-regulation in AML patients compared with the normal controls both at the mRNA (Fig. 2b) and protein (Fig. 2c) levels, and also remarkable time-course up-regulation during PMA-induced moncytic differentiation of THP-1 cells (Fig. 2d) and HL-60 cells (Fig. 2e) as well as *in vitro* monocytic induction culture of CD34^+^ HSPCs (Fig. 2f), all in accordance with array data analyzed before. Collectively, the results imply that ZFP36L1 may function as a crucial regulator in monocyte/macrophage differentiation.

### ZFP36L1 mediates PMA-induced monocyte/macrophage differentiation

It has been reported that ZFP36 family members (ZFP36L1, ZFP36 and ZFP36L2) share the homological domain and have redundant functions in some specific biological processes^16^,^28^. So we first detected the mRNA levels of the ZFP36 family members in PMA-induced monocyte/macrophage differentiation of THP-1 and HL-60 cells, which showed that only ZFP36L1 displayed significant up-regulation with ZFP36 and ZFP36L2 expression changes not as remarkable as ZFP36L1 expression (data not shown), in agreement with the array data analyzed before. Therefore in the subsequent study we put all the emphasis on ZFP36L1 in monocytic differentiation.

**Figure 3:**
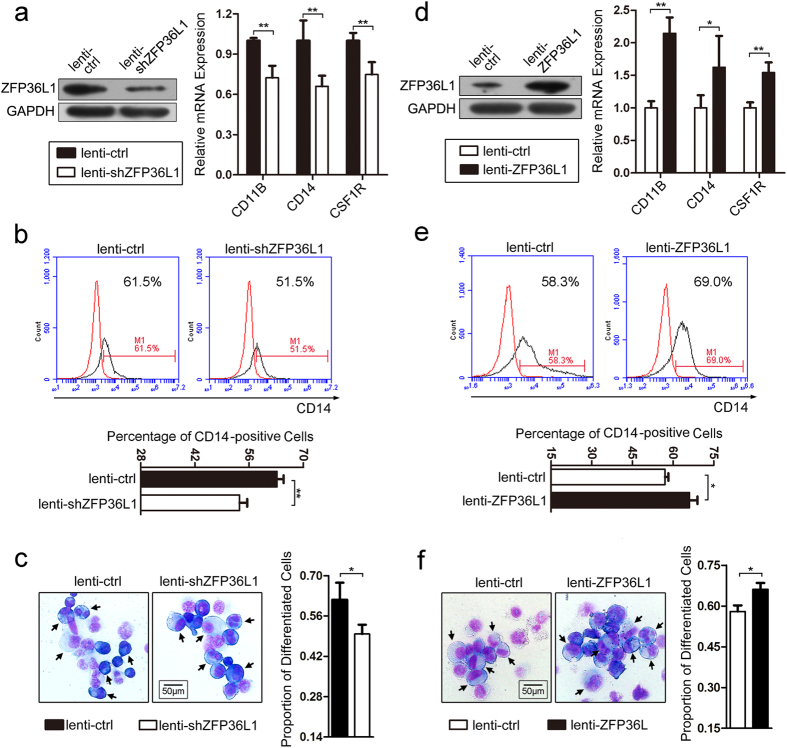
ZFP36L1 mediates PMA-induced monocyte/macrophage differentiation. (**a**) Western blot and real-time PCR analyses of expression of ZFP36L1 and the monocyte/macrophage differentiation markers CD11B, CD14 and CSF1R. THP-1 cells were infected with lenti-shZFP36L1 and lenti-ctrl respectively followed by PMA induction for 50 h. (**b**) Expression of the monocytic differentiation marker CD14 was analyzed by flow cytometry in the infected and PMA-induced cells. The red line and the black line indicate untreated cells and CD14 antibody-stained cells respectively. A representative experiment is presented in the top panel and a statistic analysis for three experiments in the bottom. Data are represented as mean ± SD. (**c**) May-Grünwald Giemsa staining in the infected and PMA-induced cells. The cells were observed under ×40 magnification. The differentiated monocyte/macrophages were indicated with arrows. A representative experiment is presented in the left. A statistical analysis of the differentiated monocyte/macrophages for counting cells in five fields is presented in the right. (**d**) Western blot and real-time PCR analyses of ZFP36L1 expression and the monocyte/macrophage differentiation markers CD11B, CD14 and CSF1R in THP-1 cells infected with lenti-ZFP36L1 and lenti-ctrl respectively followed by PMA induction for 50 h. (**e**) Expression of the monocytic differentiation marker CD14 was analyzed by flow cytometry in the lenti-ZFP36L1- or lenti-ctrl-infected and PMA-induced cells. A representative experiment is presented in the top panel and a statistic analysis for three experiments in the bottom. (**f**) May-Grünwald Giemsa staining in the lenti-ZFP36L1- or lenti-ctrl-infected and PMA-induced cells. The cells were observed under ×40 magnification. A representative experiment is presented in the left and a statistical analysis of the differentiated monocyte/macrophages for counting cells in five fields is presented in the right. *P < 0.05 and **P < 0.01, Student’s t-test.

To investigate the effect of ZFP36L1 on the differentiation, we make use of the recombined lentiviruses that express specific short hairpin RNA for ZFP36L1 (lenti-shZFP36L1) or ZFP36L1 (lenti-ZFP36L1) to infect THP-1 cells followed by PMA induction for 50 h. Western blot analysis revealed that lenti-shZFP36L1 infection remarkably decreased ZFP36L1 expression which resulted in significant down-regulation of the mRNA levels of the monocyte/macrophage differentiation markers (CD11B, CD14 and CSF1R) (Fig. 3a) as compared with the lenti-control (lenti-ctrl) infection. Flow cytometry analysis revealed decreased CD14 expression upon lenti-shZFP36L1 infection relative to lenti-ctrl infection (Fig. 3b). Besides, May-Grünwald-Giemsa staining demonstrated that lenti-shZFP36L1 infection also exhibited less proportion of differentiated monocyte/macrophages compared with the lenti-ctrl infection (Fig. 3c). These results demonstrated that knock-down of ZFP36L1 in THP-1 cells impaired PMA-induced moncyte/macrophage differentiation.

On the other hand, enforced expression of *ZFP36L1* in THP-1 cells facilitated PMA-induced monocytic differentiation. Compared with the lenti-ctrl-infected THP-1 cells, the lenti-ZFP36L1-infected cells exhibited significant over-expression of ZFP36L1 which increased mRNA levels of the monocyte/macrophage differentiation markers (CD11B, CD14 and CSF1R) (Fig. 3d). Besides, the over-presence of ZFP36L1 resulted in elevated CD14 expression detected by flow cytometry (Fig. 3e) and higher proportion of differentiated monocyte/macrophages (Fig. 3f).

Collectively, these results suggested ZFP36L1’s function as an important positive regulator in monocyte/macrophage differentiation.

### Validation of ZFP36L1’s role in monocyte/macrophage differentiation of CD34^+^ HSPCs

**Figure 4:**
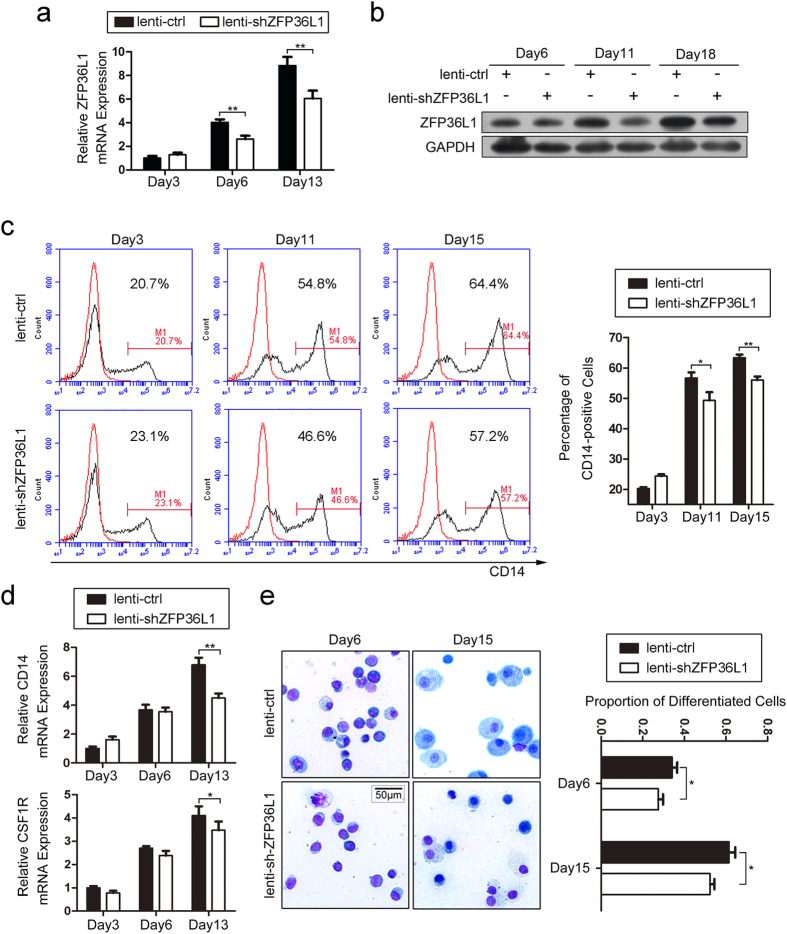
Validation of ZFP36L1’s role in monocyte/macrophage differentiation of CD34^+^ HSPCs. (**a,b**) The expression of ZFP36L1 was analyzed using real-time PCR (**a**) and western blot (**b**) during the *in vitro* monocytic induction culture of CD34^+^ HSPCs infected with lenti-shZFP36L1 or lenti-ctrl. (**c**) Expression of the monocytic differentiation marker CD14 was evaluated by flow cytometry after the infection and induction of CD34^+^ HSPCs. A representative experiment is presented in the left and the results from two independent experiments were statistically analyzed and presented as mean ± SD in the right. The red line and the black line indicate untreated cells and CD14 antibody-stained cells respectively. (**d**) Real-time PCR analysis of CD14 and CSF1R mRNA expression during the differentiation of CD34^+^ HSPCs infected with lenti-shZFP36L1 or lenti-ctrl. (**e**) May-Grünwald Giemsa staining of the infected and differentiation-induced CD34^+^ HSPCs. The cells were observed under ×40 magnification. A representative field for each experiment is presented in the left and a statistical analysis of the differentiated monocyte/macrophages by counting cells in five fields is presented in the right. Data are represented as mean ± SD. *P < 0.05 and **P < 0.01, Student’s t-test.

To verify the function of ZFP36L1 in monocyte/macrophage differentiation of HSPCs, we infected CD34^+^ HSPCs with lenti-shZFP36L1 or lenti-ctrl and then carried out *in vitro* induction culture towards monocyte/macrophage differentiation for 20 days. The lenti-shZFP36L1 infection significantly decreased ZFP36L1 expression as detected by real-time PCR (Fig. 4a) and western blot (Fig. 4b). Knock-down of ZFP36L1 in CD34^+^ HSPCs impaired monocyte/macrophage differentiation as revealed by reduced CD14 expression evaluated through flow cytometry (Fig. 4c), decreased CD14 and CSF1R mRNA levels evaluated through real time PCR (Fig. 4d), and decreased proportion of differentiated monocyte/macrophages evaluated through May-Grünwald Giemsa staining (Fig. 4e). These results further verified ZFP36L1’s important role in monocyte/macrophage development.

### CDK6 mRNA is identified as a direct target of ZFP36L1

**Figure 5:**
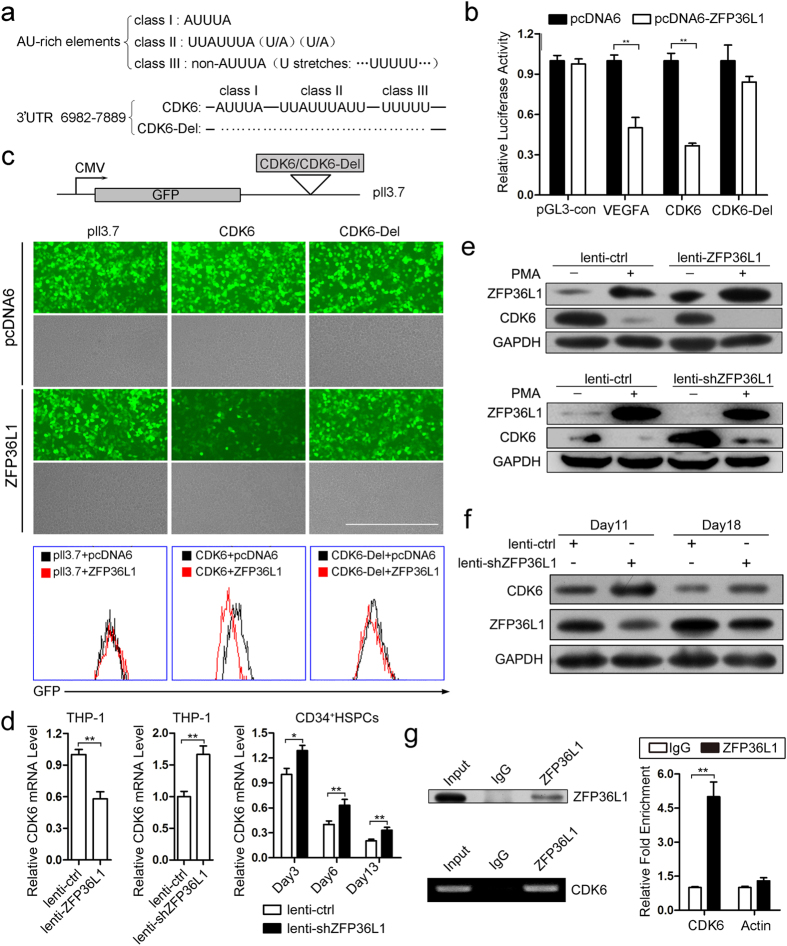
CDK6 mRNA is verified as a direct target of ZFP36L1. (**a**) Schematic outline of AU-rich elements. (**b**) Luciferase reporter assays. 293 TN cells were co-transfected with each pGL3-control-based constructs (pGL3-con, VEGFA, CDK6, CDK6-Del) and pcDNA6-ZFP36L1 (or pcDNA6). VEGFA 3′UTR was used as a positive control. Three independent experiments were performed and data are presented as mean ± SD. (**c**) GFP reporter assay. 293 TN cells were co-transfected with each pll3.7-based constructs (pll3.7, CDK6 and CDK6-Del) and pcDNA6-ZFP36L1 (or pcDNA6).The relative GFP expression was presented as fluorescence pictures (top) and also analyzed by flow cytometry (bottom). (**d**) The mRNA level of CDK6 was determined in THP-1 cells infected with lenti-ZFP36L1 (or lenti-ctrl) (left) and lenti-shZFP36L1 (or lenti-ctrl) (middle) respectively as well as during the monocytic differentiation of CD34^+^ HSPCs infected with lenti-shZFP36L1 (or lenti-ctrl) (right). (**e**) The expression of ZFP36L1 and CDK6 was analyzed by western blot in the THP-1 cells that were infected with lenti-ZFP36L1 (or lenti-ctrl) and lenti-shZFP36L1 (or lenti-ctrl) respectively, and encountered PMA induction. (**f**) Western blot analysis of ZFP36L1 and CDK6 expression in the monocytic induction cultures of CD34^+^ HSPCs infected with lenti-shZFP36L1 (or lenti-ctrl). (**g**) Immunoprecipitation using anti-ZFP36L1 or anti-IgG antibody and extracts of PMA-induced THP-1 cells. ZFP36L1 in immunoprecipitates was analyzed by immunoblot (left: top panel). RNA levels in immunoprecipitates were determined by semi-quantitative PCR (left: bottom panel) and real-time PCR (right panel). The levels of CDK6 and β-actin are presented as fold enrichment in anti-ZFP36L1 relative to anti-IgG immunoprecipitates. *P < 0.05 and **P < 0.01, Student’s t-test.

ZFP36L1 is a RNA binding protein that mainly binds the AREs in the 3′UTR and promotes the decay of the target RNAs^29^. To search for down-stream targets of ZFP36L1, we first consulted the AREsite^30^ and downloaded the mRNAs bearing UUAUUUAUU motif in their 3′UTRs, which is implicated by previous work that ZFP36L2 binds to UUAUUUAUU motif with priority^31^. There are 1393 mRNAs containing UUAUUUAUU motif. We first focused on CDK6 that has been reported to be down-regulated during myeloid differentiation^32^. Besides, CDK6 mRNA has 10 kb long 3′UTR containing numerous AREs with all three classes of AREs^33^ included. The three classes of AREs were depicted as Fig. 5a, and the fragment**s** of CDK6 3′UTR (6982–7889 bp) containing all the three classes of AREs and its deleted form (CDK6-Del) were cloned into pGL3-control and pll3.7 for subsequent experiments. Luciferase assay was performed to demonstrate that ZFP36L1 could negatively regulate CDK6-mediated luciferase expression and has less effect on CDK6-Del-directed luciferase activity, with VEGFA used as a positive control (Fig. 5b). We also conducted another experiment using GFP-based system. The GFP expression was presented as fluorescence pictures and flow cytometry analysis (Fig. 5c), which demonstrated that ZFP36L1 negatively regulates CDK6 expression in an ARE-site dependent manner. The influence of ZFP36L1 on CDK6 mRNA level was determined in THP-1 cells infected with lenti-ZFP36L1 (or lenti-ctrl) and lenti-shZFP36L1 (or lenti-ctrl) respectively as well as during the monocytic differentiation of CD34^+^ HSPCs infected with lenti-shZFP36L1 (or lenti-ctrl). The results demonstrated that ZFP36L1 negatively regulates CDK6 mRNA level both in THP-1 cells and in the time-course differentiation of CD34^+^ HSPCs (Fig. 5d). Negative regulation of ZFP36L1 on CDK6 protein level was also verified during monocyte/macrophage differentiation of THP-1 cells and CD34^+^ HSPCs. THP-1 cells were infected with lenti-ZFP36L1 (or lenti-ctrl) and lenti-shZFP36L1 (or lenti-ctrl) and then induced towards monocytic differentiation by PMA. Compared with lenti-ctrl infection, lenti-ZFP36L1 and lenti-shZFP36L1 infection significantly increased and decreased ZFP36L1 protein level respectively (Fig. 5e), which led to the remarkable down-regulation and up-regulation of CDK6 expression respectively (Fig. 5e). We also infected CD34^+^ HSPCs with lenti-shZFP36L1 or lenti-ctrl, followed by induction culture towards monocyte/macrophage differentiation. Then the protein levels of ZFP36L1 and CDK6 were detected by western blot and the same results were observed with that in THP-1 cells (Fig. 5f). To further demonstrate the direct interaction between ZFP36L1 protein and CDK6 mRNA, RNA immunoprecipitation (RIP) was carried out using antibody against ZFP36L1 and extracts of PMA-induced THP-1 cells. The specificity of ZFP36L1 antibody was confirmed by immunoprecipitation (IP) and immunoblotting (Fig. 5g, left and top panel). As revealed by the RIP semi-quantitative PCR (Fig. 5g, left and bottom panel) and RIP real-time PCR (Fig. 5g, right panel), CDK6 mRNA was preferentially enriched in ZFP36L1-containing RNPs relative to control IgG immunoprecipitates. All these results demonstrated that ZFP36L1 could bind to the AREs of CDK6 3′UTR and negatively regulate CDK6 expression.

### CDK6 impairs *in vitro* monocyte/macrophage differentiation of CD34^+^ HSPCs

**Figure 6:**
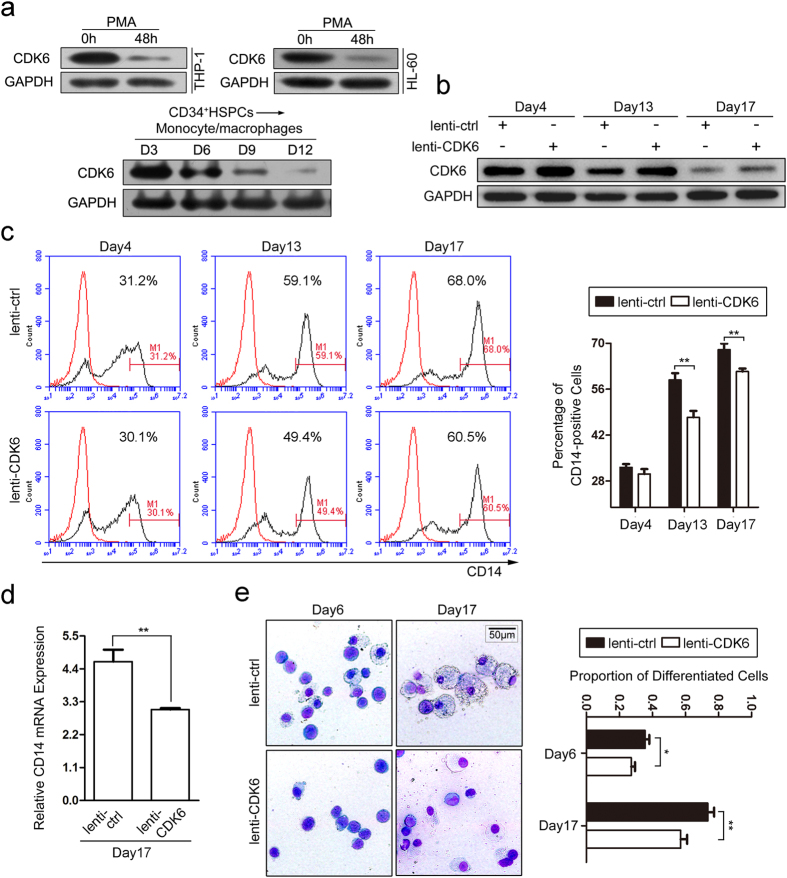
CDK6 impairs monocyte/macrophage differentiation of CD34^+^ HSPCs. (**a**) The expression of CDK6 was detected by western blot during PMA-induced THP-1 and HL-60 cells as well as monocytic differentiation of CD34^+^ HSPCs. (**b**) Western blot analysis of CDK6 expression in monocyte/macrophage induction cultures of CD34^+^ HSPCs infected with lenti-CDK6 and lenti-ctrl. (**c**) Expression of the monocytic differentiation marker CD14 was evaluated by flow cytometry after the infection and induction of CD34^+^ HSPCs. A representative experiment is presented in the left and the results from two independent experiments were statistically analyzed and presented as mean ± SD in the right. The red line and the black line indicate untreated cells and CD14 antibody stained cells respectively. (**d**) Real-time PCR analysis of CD14 mRNA expression at 17 days of differentiation of CD34^+^ HSPCs infected with lenti-CDK6 and lenti-ctrl. (**e**) May-Grünwald Giemsa staining of the infected and differentiation-induced CD34^+^ HSPCs. The cells were observed under ×40 magnification. The proportion of differentiated monocyte/macrophages was counted. Data are represented as mean ± SD. *P < 0.05 and **P < 0.01, Student’s t-test.

To investigate the effect of CDK6 on monocyte/macrophage differentiation, we first detected the expression of CDK6 in PMA induced monocytic differentiation of THP-1 and HL-60 cells as well as *in vitro* monocytic induction culture of CD34^+^ HSPCs. The results exhibited remarkable decrease of CDK6 expression during the differentiation (Fig. 6a). Then the CD34^+^ HSPCs were infected with lenti-CDK6 or lenti-ctrl and induced towards monocyte/macrophage differentiation for 20 days. Compared with lenti-ctrl infection, lenti-CDK6 infection significantly increased CDK6 protein level (Fig. 6b). The over-presence of CDK6 impaired monocytic differentiation as revealed by decreased CD14 expression detected by flow cytometry (Fig. 6c) and real-time PCR (Fig. 6d) and less proportion of differentiated monocyte/macrophages (Fig. 6e). The results imply that CDK6 functions as a repressive regulator of monocyte/macrophage differentiation.

### ZFP36L1 regulates monocyte/macrophage differentiation by targeting CDK6

**Figure 7:**
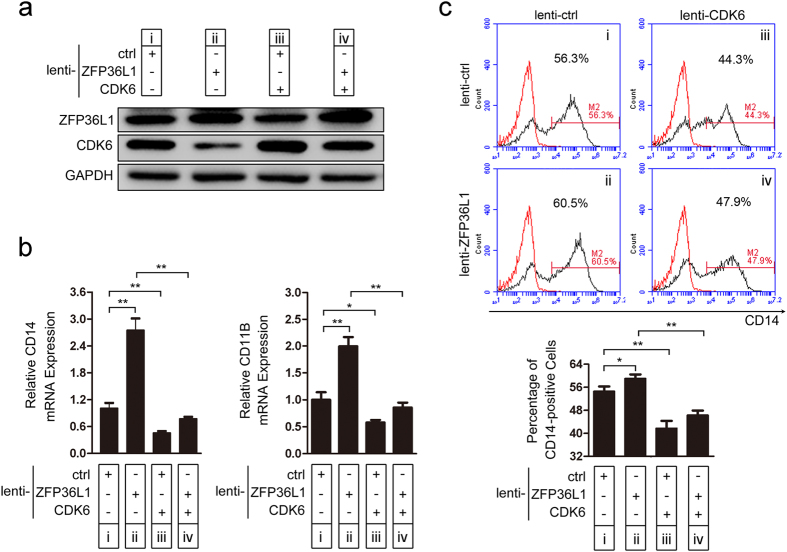
ZFP36L1 regulates monocyte/macrophage differentiation by targeting CDK6. Rescue assays were performed by infection with a combination of lenti-ZFP36L1 (or lenti-ctrl) and lenti-CDK6 (or lenti-ctrl) in CD34^+^ HSPCs followed by monocytic induction for 20 days. The cells in 16 days of differentiation were collected for further analyses. (**a**) The levels of ZFP36L1 and CDK6 were determined by western blot. (**b**) Real-time PCR of CD14 and CD11B mRNA expression. (**c**) CD14 expression was evaluated through flow cytometry analysis and a statistical analysis of two independent experiments is shown in the bottom panel. The red line and the black line indicate untreated cells and CD14 antibody stained cells respectively. *P < 0.05 and **P < 0.01, Student’s t-test.

To further confirm that ZFP36L1 regulates monocyte/macrophage differentiation through CDK6, we performed rescue assay. CD34^+^ HSPCs were infected with lenti-ZFP36L1 or lenti-ctrl. Twenty-four hours later, the cells were re-infected with lenti-CDK6 or lenti-ctrl followed by induction towards monocyte/macrophage differentiation. As expected, re-infection with lenti-CDK6 alleviated the down-regulation of CDK6 expression resulted from lenti-ZFP36L1 treatment (Fig. 7a, iv versus ii). Consistent with the CDK6 expression, re-infection with lenti-CDK6 impeded the facilitation of monocyte/macrophage differentiation caused by lenti-ZFP36L1 infection, which is presented as CD14 and CD11B mRNA expression (Fig. 7b, iv versus ii) and CD14 expression evaluated through flow cytometry (Fig. 7c, iv versus ii). These results also demonstrated that CDK6 functions directly downstream of ZFP36L1 and has stronger effect on monocyte/macrophage differentiation, which can be explained by the fact that CDK6 may act as a downstream effecter and has more than one upstream regulator, such as miR 29a^32^.

## Discussion

Hematopoiesis is an elaborately controlled process wherein the pluripotent self-renewing hematopoietic stem cells give rise to all blood cell lineages^24^. Increasing evidence suggests that this process is regulated by a complex regulatory network composed of lineage-specific transcription factors, cytokines, noncoding RNAs and RBPs^34^,^35^,^36^,^37^. Aberrant expression of any of the above regulators would lead to hematopoietic disorders and even leukemia. With the development of transcriptome sequencing, it is widely acknowledged that the mammalian genome is extensively transcribed, giving rise to thousands of noncoding transcripts which have attracted a lot of attention for their versatile regulatory role in multiple biological processes^38^,^39^. Analogous to DNA, which is organized and packed via strong associations with histones in the nucleus, RNA can’t exist alone in cells, but are stably assembled with many RBPs and other proteins to form RNPs. RBPs are essential players in RNA metabolism and regulate RNA splicing, localization, surveillance, decay and translation^40^. RBPs, as RNA partners and crucial post-transcriptional regulators, have been reported to participate in various physiological and pathological processes, including hematopoiesis and leukemogenesis^41^,^42^,^43^. Here in this study, we systematically screened the potential RBPs through bioinformatics analysis of the array data in AML patients and myeloid differentiation. Finally we focused on RBP-ZFP36L1 which was demonstrated to facilitate monocyte/macrophage differentiation. Monocyte/macrophages, whose biogenesis is an important branch of myeloid differentiation, are important immune cells to mediate inflammatory reaction and resist pathogen infection^44^.

AREs are a group of loosely defined AU-rich instability determinants with sizes ranging from 50 to150 nucleotides (nt) found in the 3′UTR of labile mRNAs which code for the regulators of cell growth, survival and differentiation, such as cytokines, proto-oncogenes and nuclear transcription factors^33^. AREs are classified into at least three classes according to the core motif: (1) class I– AUUUA (2) class II– UUAUUUA(U/A)(U/A) (3) class III – U stretches such as (…UUUUU…), all of which function in combination to determine the fate of the host mRNAs^45^. ZFP36L1 belongs to AU-rich binding proteins (AUBPs) which also include other proteins such as ELAVL1, AUF1, KHSRP and Nucleolin (NCL)^46^. AUBPs bind to the AREs leading to the target decay^47^ or stabilization^48^. Besides, AUBPs could also competitively or corporately bind the cis-element in the 3′UTR with miRNAs to make the post-transcriptional regulatory network much more complex and elaborate. Tominaga, K. *et al.* reported that HuR and miR-494 functionally competed to bind NCL 3′UTR and modulate NCL expression^49^. ZFP36 was demonstrated to be indispensable for miR-16-mediated ARE-RNA degradation^50^. HuR and let-7 were also shown to repress c-Myc expression in an interdependent manner^51^. In this study, CDK6 mRNA containing all the three classes of AREs was identified to associate with ZFP36L1 protein and CDK6 expression was negatively regulated by ZFP36L1.

AML is a kind of disease characterized by a set of gene mutations and chromosome rearrangements^52^. The influenced genes can be divided into two classes. One class includes genes related to cell differentiation, such as HOXA9, AML1, MLL and RAR α, and the other consists of genes related to proliferation or survival of cells and includes FLT3, ABL, RAS, KIT and CDK6^53^. *CDK6* encodes a kinase as a catalytic subunit of the protein kinase complex to regulate the G1 phase progression and G1/S transition of the cell cycle^54^. In mammalian cells, cell cycle is activated by CDK6 in the early G1 phase through interactions with cyclins D1, D2 and D3^55^. CDK6 correlates with cell proliferation and is often aberrantly expressed in cancers like lymphoma, leukemia, medulloblastoma and melanoma associated with chromosome rearrangements^56^,^57^. Besides, increasing evidence suggests that CDK6 may carry out additional functions independent of its kinase activity^58^. In mutant knockout mice of CDK6, the hematopoietic function is impaired, regardless of otherwise organ normal development, which might hint additional roles of CDK6 in the development of blood components^59^. Here we further demonstrated that CDK6 acts as a negative regulator to impede the *in vitro* monocyte/macrophage differentiation of CD34^+^ HSPCs.

In summary, our results demonstrated that ZFP36L1-mediated post-transcriptional control of CDK6 expression through binding to its 3′UTR implicates a novel regulatory circuit in monocyte/macrophage differentiation, which may also provide a therapeutic target for AML patients with abnormal expression of ZFP36L1 and CDK6.

## Methods

### Bioinformatics analysis

Combined with RBPDB, array data of AML patients and myeloid differentiation were used to screen the potential RBPs involved in myeloid differentiation. GSE30285, GSE34184, GSE12803 and GSE24759 were downloaded from GEO DataSets and analyzed using bioinformatics tools developed by a member in our lab.

### Human samples

Human UCB was obtained from normal full-term deliveries from Beijing Hospital. The peripheral blood samples of AML patients and normal volunteers were obtained from the 303 hospital and the Beijing 307 Hospital. The informed consent to perform the biological studies was obtained from all of the examined subjects and the related study was approved by the Ethic Committees of the Institutional Review Board of IBMS, CAMS. The methods were carried out in accordance with the approved guidelines. Mononuclear cells (MNCs) fractions were isolated from the samples by Percoll density gradient [d = 1.077 g/ml], (Amersham Biotech, Germany) and CD34^+^ cells were enriched from MNCs through positive immunomagnetic selection (CD34 MultiSort kit, Miltenyi Biotech, Bergisch-Glad-bach, Germany).

### Cell culture and differentiation induction

The following human cell lines were used in this study: THP-1 and HL-60 purchased from cell resource center of Shanghai Institutes for Biological Science, 293 TN purchased from cell resource center of Institutes of Basic Medical Sciences, Chinese Academy of Medical Sciences. THP-1 was cultured in PRMI 1640 medium (Gibco); HL-60 was cultured in Iscove’s Modified Dubecco’s Medium (IMDM) (Gibco, BRL, UK); 293 TN was cultured in Dulbecco’s modified Eagle’s medium (DMEM) (Gibco, BRL, UK). All cultures were supplemented with 10% fetal bovine serum (FBS) (Hyclone), 100 U/ml penicillin and 100 μg/ml streptomycin (Sigma-Aldrich, St. Louis, Mo, USA) at 37 °C in 5% CO_2_. The monocyte/macrophage differentiation of THP-1 and HL-60 was induced with PMA (Sigma-Aldrich) at final concentration of 10 nM. The monocyte/macrophage differentiation culture of CD34^+^ HSPCs was performed as described previously^32^.

### RNA extraction, reverse transcription and quantitative real time PCR

Total RNA was extracted from the cell samples using the Trizol reagent (Invitrogen, CA, USA) according to manufacturer’s instructions. 1~2 μg of total RNA was used to generate cDNA by M-MLV reverse transcriptase (Invitrogen, Carlsbad, CA, USA). Oligo(dT)_18_ was used for reverse transcription of mRNAs. Quantitative real-time PCR was carried out using Bio-Rad CFX-96 (Bio-Rad, CA, USA) in triplicate. The data were normalized to GAPDH mRNA expression. The following primers for real-time PCR were used: ZFP36L1, F: ATGACCACCACCCTCGTGT, R: TTTCTGTCCAGCAGGCAACC; CDK6, F1: GGTCAGGTTGTTTGATGTGTGC, R1: TCGGTGTGAATGAAGAAA GTCC, F2: GAACAGCACCTGACAGGCG, R2: GCAGCTTATTTGGGGGCTTAG TC; CD14, F: CGCTCCGAGATGCATGTG, R: TTGGCTGGCAGTCCTTTAGG; CD11B, F: GGGCTGGTGGAGTCTTTCTAT, R: TTCTGCCTGAACATCGCTA; CSF1R, F: CCTGAAGGTGGCTGTGAAGATG, R: GCTCCCAGAAGGTTGACGATG; GAPDH, F: GGAGCGAGATCCCTCCAAAAT, R: GGCTGTTGTCATACTTCTCATGG; Actin, F: CTGGCACCACACCTTCTACA, R: AGCACAGCCTGGATAGCAAC.

### Plasmid construction

The ZFP36L1 cDNA was amplified and cloned into pmiRNA1 (System Biosciences, SBI) and pcDNA6 (Invitrogen) to get its expression plasmids. The cDNA of CDK6 was amplified and cloned into pmiRNA1 (System Biosciences, SBI) to realize its over-expression. The fragment of CDK6 3′UTR and its deleted form was inserted into luciferase reporter vector pGL3-control (Promega) and pll3.7 (Addgene). The short hairpin RNA (shRNA) sequences for ZFP36L1 and non-target control were synthesized, annealed and inserted into pll3.7 (Addgene). The following primers and oligonucleotides for plasmid construction were used: ZFP36L1, CEF1: GAATTCCGAACGCACAGGATGA (EcoR I), CER1: GCGGCCGCACCTTGTTA ATGTAGG (Not I); CDK6, CEF1: GAATTCGCGTCCAGGCGGCATGG (EcoR I), CER1: GGATCCTGAGGCCTCAGGCTGTA (BamH I); CDK6, EF1: TCTAGAGCACACATAGAGCCACACAA (Xba I), ER1: GGCCGGCCATGAGG GCAGACAAGAG (Fse I); VEGFA, EF1: TCTAGAACAGAGAGACAGGGCAGG (Xba I), ER1: GGCCGGCCAATATCTCGAAAAACTG (Fse I); ZFP36L1-shRNA, F: TGTAACAAGATGCTCAACTATTCAAGAGATAGTTGAGCATCTTGTTACT TTTTTC, R: TCGAGAAAAAAGTAACAAGATGCTCAACTATCTCTTGAATAG TTGAGCATCTTGTTACA; control-shRNA, F: TGAACTCAAGACCGATATTATT CAAGAGATAATATCGGTCTTGAGTTCTTTTTTC, R: TCGAGAAAAAAGAAC TCAAGACCGATATTATCTCTTGAATAATATCGGTCTTGAGTTCA.

### Luciferase reporter assay

293 TN cells were co-transfected with pGL3-control constructs, pRL-TK and pcDNA-ZFP36L1 (or pcDNA6) using Lipofectamine 2000 (Invitrogen) in 24-well plate. The plasmid pRL-TK containing Renila luciferase was used as an internal control. The transfection medium was replaced with complete medium after 5–6 h. The cells were cultured at 37 °C in 5% CO_2_ for an additional 24–48 h. The cells were harvested and the luciferase activity was measured using dual luciferase assay system (Promega) according to the manufacturer’s instructions.

### Western blot

Cell lysates were subjected to SDS/PAGE (10% separation gel) and transferred onto a PVDF membrane. Primary antibodies against the following proteins were used: CDK6 (ab124821, Abcam), ZFP36L1 (BS3004, Bioworld), ZFP36L1 (ABN192, Millipore) and GAPDH (10494-1-AP, Proteintech). Horseradish peroxidase conjugated secondary antibodies were used. Signals were detected using an ECL (enhanced chemiluminescence) kit (Millipore).

### Lentivirus production and cell infection

The recombination lentiviruses for over-expression and knock-down were produced using the pmiRNA1- and pll3.7-based constructs. Lentivirus packaging was performed using the pPACKH1^TM^ Lentiviral Vector Packaging Kit (LV500A-1, System Biosciences, SBI, CA, USA) according to the manufacturer’s instructions. The virus particles were condensed using the PEG-it™ Virus Precipitation Solution (SBI, CA, USA). The THP-1 cells and CD34^+^ HSPCs were infected with lentivirus in 6-well plates containing 5 μg/ml polybrene (Sigma Aldrich). After 24 h infection, the cells were replaced with fresh complete medium and induced towards monocyte/macrophage differentiation.

### RIP

THP-1 cells were plated onto 10 cm plates and grown to approximately 80% confluences. Then the cells were induced towards monocyte/macrophage differentiation for 48 h and RIP was performed using the Magna RIP™ RNA-Binding Protein Immunoprecipitation Kit (17–700, Millipore) according to the manufacturer’s instructions. Primary antibodies against the following proteins were used: ZFP36L1 (ABN192, Millipore), rabbit IgG (PP64B, Millipore).

### Flow cytometry analysis

The infected THP-1 cells and the CD34^+^ HSPCs were induced towards monocyte/macrophage differentiation and harvested at different time points of differentiation. The cells were rinsed twice with PBS and re-suspended in 100 μl PBS. Then the cells were incubated with PE/APC-conjugated anti-CD14 (eBioscience) at 4 °C for 30 min. Then the cells were washed with 1 ml PBS, re-suspended in 200 μl PBS and analyzed immediately using an AccuriC6 flow cytometer (BD, SD, USA). 293TN cells co-transfected with pll3.7-CDK6 (or pll3.7-CDK6-Del or pll3.7) and pcDNA6-ZFP36L1 (or pcDNA6) were collected, rinsed twice with PBS, re-suspended in 200 μl PBS and analyzed immediately using an AccuriC6 flow cytometer (BD, SD, USA).

### May-Grünwald Giemsa staining

The THP-1 cells or CD34^+^ HSPCs cells induced towards monocyte/macrophage differentiation were harvested at different time points of differentiation and stained with May-Grünwald for 5 min and Giemsa for 20 min. The cell smears were washed with distilled water, air-dried, and observed under optical microscopy Olympus BX51 (Olympus, Tokyo, Japan).

### Statistical Analysis

Student’s t-test (two-tailed) was performed to analyze the data. Statistical significance was set at *P* < 0.05, as indicated by an asterisk (*P < 0.05; **P < 0.01).

## Additional Information

**How to cite this article**: Chen, M.-T. *et al.* ZFP36L1 promotes monocyte/macrophage differentiation by repressing CDK6. *Sci. Rep.* **5**, 16229; doi: 10.1038/srep16229 (2015).

## References

[b1] Vogel C. *et al.* Sequence signatures and mRNA concentration can explain two-thirds of protein abundance variation in a human cell line. Mol Syst Biol 6, 400, 10.1038/msb.2010.59 (2010). 20739923 PMC2947365

[b2] Ye J. & Blelloch R. Regulation of pluripotency by RNA binding proteins. Cell Stem Cell 15, 271–280, 10.1016/j.stem.2014.08.010 (2014). 25192462 PMC4372238

[b3] Weake V. M. *et al.* Post-transcription initiation function of the ubiquitous SAGA complex in tissue-specific gene activation. Genes Dev 25, 1499–1509, 10.1101/gad.2046211 (2011). 21764853 PMC3143940

[b4] Castello A. *et al.* Insights into RNA biology from an atlas of mammalian mRNA-binding proteins. Cell 149, 1393–1406, 10.1016/j.cell.2012.04.031 (2012). 22658674

[b5] Ciafre S. A. & Galardi S. microRNAs and RNA-binding proteins: a complex network of interactions and reciprocal regulations in cancer. RNA Biol 10, 935–942, 10.4161/rna.24641 (2013). 23696003 PMC4111733

[b6] Bartel D. P. MicroRNAs: genomics, biogenesis, mechanism, and function. Cell 116, 281–297, S0092867404000455 (2004). 14744438 10.1016/s0092-8674(04)00045-5

[b7] Lunde B. M., Moore C. & Varani G. RNA-binding proteins: modular design for efficient function. Nat Rev Mol Cell Biol 8, 479–490, 10.1038/nrm2178 (2007). 17473849 PMC5507177

[b8] Cook K. B., Kazan H., Zuberi K., Morris Q. & Hughes T. R. RBPDB: a database of RNA-binding specificities. Nucleic Acids Res 39, D301–308, 10.1093/nar/gkq1069 (2011). 21036867 PMC3013675

[b9] Glisovic T., Bachorik J. L., Yong J. & Dreyfuss G. RNA-binding proteins and post-transcriptional gene regulation. FEBS Lett 582, 1977–1986, 10.1016/j.febslet.2008.03.004 (2008). 18342629 PMC2858862

[b10] Li J. H., Liu S., Zhou H., Qu L. H. & Yang J. H. starBase v2.0: decoding miRNA-ceRNA, miRNA-ncRNA and protein-RNA interaction networks from large-scale CLIP-Seq data. Nucleic Acids Res 42, D92–97, 10.1093/nar/gkt1248 (2014). 24297251 PMC3964941

[b11] Zhang X. *et al.* RAID: a comprehensive resource for human RNA-associated (RNA-RNA/RNA-protein) interaction. RNA 20, 989–993, 10.1261/rna.044776.114 (2014). 24803509 PMC4114696

[b12] Li Y. *et al.* ViRBase: a resource for virus-host ncRNA-associated interactions. Nucleic Acids Res 43, D578–582, 10.1093/nar/gku903 (2015). 25274736 PMC4384010

[b13] Blackshear P. J. *et al.* Zfp36l3, a rodent X chromosome gene encoding a placenta-specific member of the Tristetraprolin family of CCCH tandem zinc finger proteins. Biol Reprod 73, 297–307, 10.1095/biolreprod.105.040527 (2005). 15814898

[b14] Frederick E. D., Ramos S. B. & Blackshear P. J. A unique C-terminal repeat domain maintains the cytosolic localization of the placenta-specific tristetraprolin family member ZFP36L3. J Biol Chem 283, 14792–14800, 10.1074/jbc.M801234200 (2008). 18367448 PMC2671328

[b15] Ciais D., Cherradi N. & Feige J. J. Multiple functions of tristetraprolin/TIS11 RNA-binding proteins in the regulation of mRNA biogenesis and degradation. Cell Mol Life Sci 70, 2031–2044, 10.1007/s00018-012-1150-y (2013). 22968342 PMC11113850

[b16] Baou M., Jewell A. & Murphy J. J. TIS11 family proteins and their roles in posttranscriptional gene regulation. J Biomed Biotechnol 2009, 634520, 10.1155/2009/634520 (2009). 19672455 PMC2722025

[b17] Ciais D. *et al.* Destabilization of vascular endothelial growth factor mRNA by the zinc-finger protein TIS11b. Oncogene 23, 8673–8680, 10.1038/sj.onc.1207939 (2004). 15467755

[b18] Zekavati A. *et al.* Post-transcriptional regulation of BCL2 mRNA by the RNA-binding protein ZFP36L1 in malignant B cells. PLoS One 9, e102625, 10.1371/journal.pone.0102625 (2014). 25014217 PMC4094554

[b19] Carballo E., Lai W. S. & Blackshear P. J. Feedback inhibition of macrophage tumor necrosis factor-alpha production by tristetraprolin. Science 281, 1001–1005, 10.1126/science.281.5379.1001 (1998). 9703499

[b20] Adachi S. *et al.* ZFP36L1 and ZFP36L2 control LDLR mRNA stability via the ERK-RSK pathway. Nucleic Acids Res 42, 10037–10049, 10.1093/nar/gku652 (2014). 25106868 PMC4150769

[b21] Kaplan I. M. *et al.* Deletion of tristetraprolin caused spontaneous reactive granulopoiesis by a non-cell-autonomous mechanism without disturbing long-term hematopoietic stem cell quiescence. J Immunol 186, 2826–2834, 10.4049/jimmunol.1002806 (2011). 21270394 PMC3114656

[b22] Stumpo D. J. *et al.* Targeted disruption of Zfp36l2, encoding a CCCH tandem zinc finger RNA-binding protein, results in defective hematopoiesis. Blood 114, 2401–2410, 10.1182/blood-2009-04-214619 (2009). 19633199 PMC2746470

[b23] Hodson D. J. *et al.* Deletion of the RNA-binding proteins ZFP36L1 and ZFP36L2 leads to perturbed thymic development and T lymphoblastic leukemia. Nat Immunol 11, 717–724, 10.1038/ni.1901 (2010). 20622884 PMC2953641

[b24] Zhu J. & Emerson S. G. Hematopoietic cytokines, transcription factors and lineage commitment. Oncogene 21, 3295–3313, 10.1038/sj.onc.1205318 (2002). 12032771

[b25] Vignudelli T. *et al.* ZFP36L1 negatively regulates erythroid differentiation of CD34+ hematopoietic stem cells by interfering with the Stat5b pathway. Mol Biol Cell 21, 3340–3351, 10.1091/mbc.E10-01-0040 (2010). 20702587 PMC2947470

[b26] Hou V. C. *et al.* Decrease in hnRNP A/B expression during erythropoiesis mediates a pre-mRNA splicing switch. EMBO J 21, 6195–6204, 10.1093/emboj/cdf625 (2002). 12426391 PMC137214

[b27] Hu W., Yuan B. & Lodish H. F. Cpeb4-mediated translational regulatory circuitry controls terminal erythroid differentiation. Dev Cell 30, 660–672, 10.1016/j.devcel.2014.07.008 (2014). 25220394 PMC4182162

[b28] Sanduja S., Blanco F. F. & Dixon D. A. The roles of TTP and BRF proteins in regulated mRNA decay. Wiley Interdiscip Rev RNA 2, 42–57, 10.1002/wrna.28 (2011). 21278925 PMC3030256

[b29] Stoecklin G. *et al.* Functional cloning of BRF1, a regulator of ARE-dependent mRNA turnover. EMBO J 21, 4709–4718, 10.1093/emboj/cdf444 (2002). 12198173 PMC126184

[b30] Gruber A. R., Fallmann J., Kratochvill F., Kovarik P. & Hofacker I. L. AREsite: a database for the comprehensive investigation of AU-rich elements. Nucleic Acids Res 39, D66–69, 10.1093/nar/gkq990 (2011). 21071424 PMC3013810

[b31] Hudson B. P., Martinez-Yamout M. A., Dyson H. J. & Wright P. E. Recognition of the mRNA AU-rich element by the zinc finger domain of TIS11d. Nat Struct Mol Biol 11, 257–264, 10.1038/nsmb738 (2004). 14981510

[b32] Wang X. S. *et al.* MicroRNA-29a and microRNA-142-3p are regulators of myeloid differentiation and acute myeloid leukemia. Blood 119, 4992–5004, 10.1182/blood-2011-10-385716 (2012). 22493297

[b33] Chen C. Y. & Shyu A. B. AU-rich elements: characterization and importance in mRNA degradation. Trends Biochem Sci 20, 465–470, 10.1016/S0968-0004(00)89102-1 (1995). 8578590

[b34] Endele M., Etzrodt M. & Schroeder T. Instruction of hematopoietic lineage choice by cytokine signaling. Exp Cell Res 329, 207–213, 10.1016/j.yexcr.2014.07.011 (2014). 25046868

[b35] Undi R. B., Kandi R. & Gutti R. K. MicroRNAs as Haematopoiesis Regulators. Adv Hematol 2013, 695754, 10.1155/2013/695754 (2013). 24454381 PMC3884629

[b36] Morlando M., Ballarino M. & Fatica A. Long Non-Coding RNAs: New Players in Hematopoiesis and Leukemia. Front Med (Lausanne) 2, 23, 10.3389/fmed.2015.00023 (2015). 25927065 PMC4396502

[b37] Chen M. T. *et al.* PU.1-Regulated Long Noncoding RNA lnc-MC Controls Human Monocyte/Macrophage Differentiation through Interaction with MicroRNA 199a-5p. Mol Cell Biol 35, 3212–3224, 10.1128/MCB.00429-15 (2015). 26149389 PMC4539372

[b38] Djebali S. *et al.* Landscape of transcription in human cells. Nature 489, 101–108, 10.1038/nature11233 (2012). 22955620 PMC3684276

[b39] Hu W., Alvarez-Dominguez J. R. & Lodish H. F. Regulation of mammalian cell differentiation by long non-coding RNAs. EMBO Rep 13, 971–983, 10.1038/embor.2012.145 (2012). 23070366 PMC3492712

[b40] Kim M. Y., Hur J. & Jeong S. Emerging roles of RNA and RNA-binding protein network in cancer cells. BMB Rep 42, 125–130, 10.5483/BMBRep.2009.42.3.125 (2009). 19335997

[b41] Ostareck-Lederer A. & Ostareck D. H. Precision mechanics with multifunctional tools: how hnRNP K and hnRNPs E1/E2 contribute to post-transcriptional control of gene expression in hematopoiesis. Curr Protein Pept Sci 13, 391–400, CPPS-EPUB-20120618-6 (2012). 22708489 10.2174/138920312801619484

[b42] Eiring A. M. *et al.* miR-328 functions as an RNA decoy to modulate hnRNP E2 regulation of mRNA translation in leukemic blasts. Cell 140, 652–665, 10.1016/j.cell.2010.01.007 (2010). 20211135 PMC2924756

[b43] Yuan J. & Muljo S. A. Exploring the RNA world in hematopoietic cells through the lens of RNA-binding proteins. Immunol Rev 253, 290–303, 10.1111/imr.12048 (2013). 23550653 PMC3620839

[b44] Shi C. & Pamer E. G. Monocyte recruitment during infection and inflammation. Nat Rev Immunol 11, 762–774, 10.1038/nri3070 (2011). 21984070 PMC3947780

[b45] Peng S. S., Chen C. Y. & Shyu A. B. Functional characterization of a non-AUUUA AU-rich element from the c-jun proto-oncogene mRNA: evidence for a novel class of AU-rich elements. Mol Cell Biol 16, 1490–1499 (1996). 8657122 10.1128/mcb.16.4.1490 PMC231133

[b46] Baou M., Norton J. D. & Murphy J. J. AU-rich RNA binding proteins in hematopoiesis and leukemogenesis. Blood 118, 5732–5740, 10.1182/blood-2011-07-347237 (2011). 21917750

[b47] Nasir A. *et al.* ZFP36L1 negatively regulates plasmacytoid differentiation of BCL1 cells by targeting BLIMP1 mRNA. PLoS One 7, e52187, 10.1371/journal.pone.0052187 (2012). 23284928 PMC3527407

[b48] Sengupta T. K., Bandyopadhyay S., Fernandes D. J. & Spicer E. K. Identification of nucleolin as an AU-rich element binding protein involved in bcl-2 mRNA stabilization. J Biol Chem 279, 10855–10863, 10.1074/jbc.M309111200 (2004). 14679209

[b49] Tominaga K. *et al.* Competitive regulation of nucleolin expression by HuR and miR-494. Mol Cell Biol 31, 4219–4231, 10.1128/MCB.05955-11 (2011). 21859890 PMC3187287

[b50] Jing Q. *et al.* Involvement of microRNA in AU-rich element-mediated mRNA instability. Cell 120, 623–634, 10.1016/j.cell.2004.12.038 (2005). 15766526

[b51] Kim H. H. *et al.* HuR recruits let-7/RISC to repress c-Myc expression. Genes Dev 23, 1743–1748, 10.1101/gad.1812509 (2009). 19574298 PMC2720259

[b52] Kelly L. M. & Gilliland D. G. Genetics of myeloid leukemias. Annu Rev Genomics Hum Genet 3, 179–198, 032802.115046 (2002). 12194988 10.1146/annurev.genom.3.032802.115046

[b53] Hirano T. *et al.* Long noncoding RNA, CCDC26, controls myeloid leukemia cell growth through regulation of KIT expression. Mol Cancer 14, 90, 10.1186/s12943-015-0364-7 (2015). 25928165 PMC4423487

[b54] Lim S. & Kaldis P. Cdks, cyclins and CKIs: roles beyond cell cycle regulation. Development 140, 3079–3093, 10.1242/dev.091744 (2013). 23861057

[b55] Meyerson M. & Harlow E. Identification of G1 kinase activity for cdk6, a novel cyclin D partner. Mol Cell Biol 14, 2077–2086, 10.1128/MCB.14.3.2077 (1994). 8114739 PMC358568

[b56] Placke T. *et al.* Requirement for CDK6 in MLL-rearranged acute myeloid leukemia. Blood 124, 13–23, 10.1182/blood-2014-02-558114 (2014). 24764564 PMC4190617

[b57] Negrini S., Gorgoulis V. G. & Halazonetis T. D. Genomic instability–an evolving hallmark of cancer. Nat Rev Mol Cell Biol 11, 220–228, 10.1038/nrm2858 (2010). 20177397

[b58] Grossel M. J. & Hinds P. W. From cell cycle to differentiation: an expanding role for cdk6. Cell Cycle 5, 266–270, 10.4161/cc.5.3.2385 (2006). 16410727

[b59] Kozar K. & Sicinski P. Cell cycle progression without cyclin D-CDK4 and cyclin D-CDK6 complexes. Cell Cycle 4, 388–391, 10.4161/cc.4.3.1551 (2005). 15738651

